# Intra- and inter-rater reproducibility of ultrasound imaging of patellar and quadriceps tendons in critically ill patients

**DOI:** 10.1371/journal.pone.0219057

**Published:** 2019-06-27

**Authors:** Joana Castro, Karina Livino de Carvalho, Paulo Eugênio Silva, Emerson Fachin-Martins, Nicolas Babault, Rita de Cássia Marqueti, João Luiz Quagliotti Durigan

**Affiliations:** 1 Graduate Program in Rehabilitation Sciences, University of Brasília (UnB), Ceilândia, Federal District, Brazil; 2 Institute of Strategic Health Management of the Federal District (IGESDF), Brasília, Brazil; 3 Graduate Program in Science and Technology in Health, University of Brasília (UnB), Ceilândia, Federal District, Brazil; 4 INSERM UMR1093-CAPS, Université Bourgogne Franche-Comté, UFR des Sciences du Sport, Dijon, France; University of Notre Dame Australia, AUSTRALIA

## Abstract

Since the outset of body image reconstruction for diagnosis purposes, ultrasound has been used to investigate structural changes located in tendons. Ultrasound has clinical applications in the intensive care unit, but its utility for tendon imaging remains unknown. Thus, we aimed to determine intra- and inter-rater reproducibility of measures obtained by images generated through morphological tendon sonographic analysis recorded from critically ill patients. We designed a cross-sectional study to assess thickness, cross-sectional area, and echogenicity of patellar and quadriceps tendons in a convenience sample formed with 20 critically ill patients. Two independent raters (experienced and novice) recorded repeated measures, checking for agreement (Kappa statistics) and reliability (Intraclass coefficient Correlation-ICC and Bland-Altman). The quality of images acquired by the two independent raters substantially agreed (k = 0.571–1.000), regardless of the region on the patellar tendon or the studied tendon (patellar or quadriceps). Regardless of how much experience the rater had, their repeated records (intra-rater reliability) always demonstrated almost complete correlation, ICC ranging from 0.89 to 0.98 for both tendons in all outcomes. At the same way, the statistically significant inter-rater ICC ranging from 0.87 to 0.97. Both repeated measures by the raters (intra-rater) and the repeated single and double measures between the raters (inter-rater) presented a minimum measurement error constituting a predominant pattern of random variability. We conclude that ultrasound imaging acquisition performed by independent raters for tendon thickness, CSA, and echogenicity monitoring of critically ill patients are acceptable and are not influenced by rater experience.

## Introduction

Critically ill patients suffer from extensive muscle wasting and atrophy, which occurs rapidly at the onset of an intensive care unit (ICU) stay [1–3]. The occurrence of systemic changes, including musculoskeletal, is well established in the literature due to prolonged immobility [4–6]. There is a reduction in tissue stiffness, cross-sectional area (CSA), and tendon thickness in disuse-induced situations [5–9]. Parry et al (2015) showed that ultrasound (US) detected changes in the quadriceps muscle correlate with strength and other health related losses observed in critically ill patients [10]. A recent study also demonstrated that only muscle area and thickness significantly decreased, without any modification in the quadriceps rectus femoris central tendon thickness in mechanically-ventilated patients [2]. Although assessment of the integrity of the tendon is fundamental in the critical care environment, to enable quadriceps actions, no studies have verified the reproducibility of measures taken by US image acquisition from the patellar and quadriceps tendons in patients admitted to the ICU.

When considering the morphological characteristics of the mentioned tendons in the ICU, imaging tests such as Magnetic Resonance Imaging (MRI) and diagnostic ultrasound are the preferred options as these techniques do not use ionizing radiation [11]. MRI is most commonly used for tendon assessment even if the choice represents an expensive and not very accessible exam [12]. For this reason, the use of high resolution US has also been indicated as an alternative exam [12,13].

Quadriceps tendon measures obtained by US have demonstrated similar accuracy to those obtained by MRI in adult patients with suspected quadriceps ruptures [12,14,15]. In addition, US measures can be recorded using portable devices, and are relatively inexpensive and easy to handle, a provident choice for assessing patients confined to bed [16,17].

The main technical limitation of US is related to the dependence on raters’ perception which could lead to measurement errors and misinterpretation [18–24]. Tiny changes or displacements coming from the ultrasonic head placement, as well as different pressure or orientation of the transducer, may significantly influence image acquisition [24]. For example, a tendon image acquired slightly oblique to the longitudinal axis of the structure may appear thicker than the image taken on its true axis [19]. The mentioned features could lead to a premature conclusion that measurements taken from US are not reliable; however, this should be systematically verified.

Reproducibility refers to the extent to which repeated measurements provide similar results from different opinions (agreement) or repeated measures (reliability) [25]. Researchers have demonstrated acceptable reproducibility for US measures of thickness and CSA of the patellar tendon in ambulatory patients, expressing intraclass correlation coefficient (ICC) ranging from 0.70–0.95 and 0.68–0.99, respectively [19–22,26].

In addition, few reports have verified ultrasound imaging in measuring tendon for patients admitted to the ICU [2]. While there is high reproducibility of ultrasound measured quadriceps tendon thickness and CSA [19,27], there is a lack of literature evaluating the reproducibility of these measures in critically ill patients. Therefore, we aimed to determine intra- and inter-rater reproducibility of thickness, CSA and echogenicity measures of tendon sonographic images recorded from critically ill patients. Our hypothesis was that US image acquisition in patients with critical illnesses would be reproducible, regardless of the rater’s experience.

## Materials and methods

An observational blinded study with repeated measures was conducted to determine the intra- and inter-rater reproducibility of measures obtained by ultrasound images from patellar and quadriceps tendons in sedated and critically ill patients admitted in the ICU of a Brazilian tertiary hospital. This study was approved by an institutional Ethics Committee of Fundação de Ensino e Pesquisa em Ciências da Saúde da Secretaria de Estado de Saúde do Distrito Federal (FEPECS / SES-DF) n° 1.768.479 in accordance with the Helsinki Declaration of 1975. Informed consent was signed by a close relative since all the patients were sedated.

### Sample

We recruited adult patients (aged ≥ 18 years) who were critically ill, sedated, and submitted to mechanical ventilation. Patients with any kind of wound on the skin where the image acquisition took place or any known change in tendon morphology (e.g., ankylosing spondylitis or rheumatoid arthritis) were excluded [19]. A prior pilot study performed with 5 patients (results unpublished) recommended a sample size of 18 participants, considering a minimally acceptable coefficient of 0.700 for an expected coefficient of 0.900, supposing type I (α = 0.05) and type II (β = 0.20) error rates [28]. Although 18 subjects were technically sufficient according to the power adopted (0.800), considering possible loss of data, we formed a convenience sample composed of 20 patients.

#### Raters

Two independent raters performed the techniques recommended for US image acquisition. Although both raters were physical therapists habituated to dealing with inpatient care, only one had experience in recording US measures, denominated as the experienced rater (KLC) for the purpose of this study. The novice rater (JC) received technical training to operate the US and acquire measures from the knee but did not have previous experience in this area. The practice consisted of a 20-minute session as described previously [29]. A basic technical explanation of the protocol and supervised performance in five patients was performed before data collection.

### Study protocol

#### Image acquisition

The patellar and quadriceps tendons of the right knee of each eligible subject were studied. US images were acquired with a SonoSite M-Turbo portable ultrasound device (Sonosite, Inc., Bothell, WA, USA), equipped with a 2-dimensional, high-frequency linear array probe (HFL38, bandwidth: 13-6MHz, maximal scan depth: 6cm). Subjects were placed in the supine position with their knees in passive extension and neutral rotation 10 minutes before the onset of recording.

Reference marks were made on the skin, taking into account the total size of the quadriceps and patellar tendons to ensure minimal placement criteria. The array probes were placed 3 cm proximal to the superior pole of the patella for the quadriceps tendon [30] and at 25%, 50%, and 75% of the length of the patellar tendon [31]. The patellar tendon length was measured between the deep insertion in the patella and the deep insertion in the tibial tuberosity [31]. These landmarks were easily visible in the ultrasonographic image as hyperechogenic regions in bone insertion.

The raters positioned the probe perpendicularly and transversally (axial plane) over the marks, maintaining constant depth, gain, and ultrasound settings throughout the data collection period. Water-soluble transmission gel was used to decrease the pressure of the transducer on the skin [32] and optimize acoustic transmission [33]. We randomized the order of rater acquisition by employing a random numerical sequence generated on the website: https://www.random.org. Each rater acquired two sequential images on the same day, without checking the first one, as the quality of images was verified off-line.

The two images (first and second records) were made by each rater in order to investigate the intra-rater reliability, with the transducer decoupled from the skin and repositioned for the next record. The data were stored as files on the device itself and subsequently transferred to a computer for off-line processing. A third blinded researcher (PES) removed the stored files identifying the image. Subsequently, the file was evaluated by the experienced rater (KLC); sixteen images per individual were collected.

#### Analysis and experimental conditions

All images were analyzed with ImageJ software (National Institute of Health, Bethesda, MD, USA), allowing the raters to classified the images, as well as record the tendon thickness, CSA, and echogenicity for both the patellar and quadriceps tendons.

**Fig 1:**
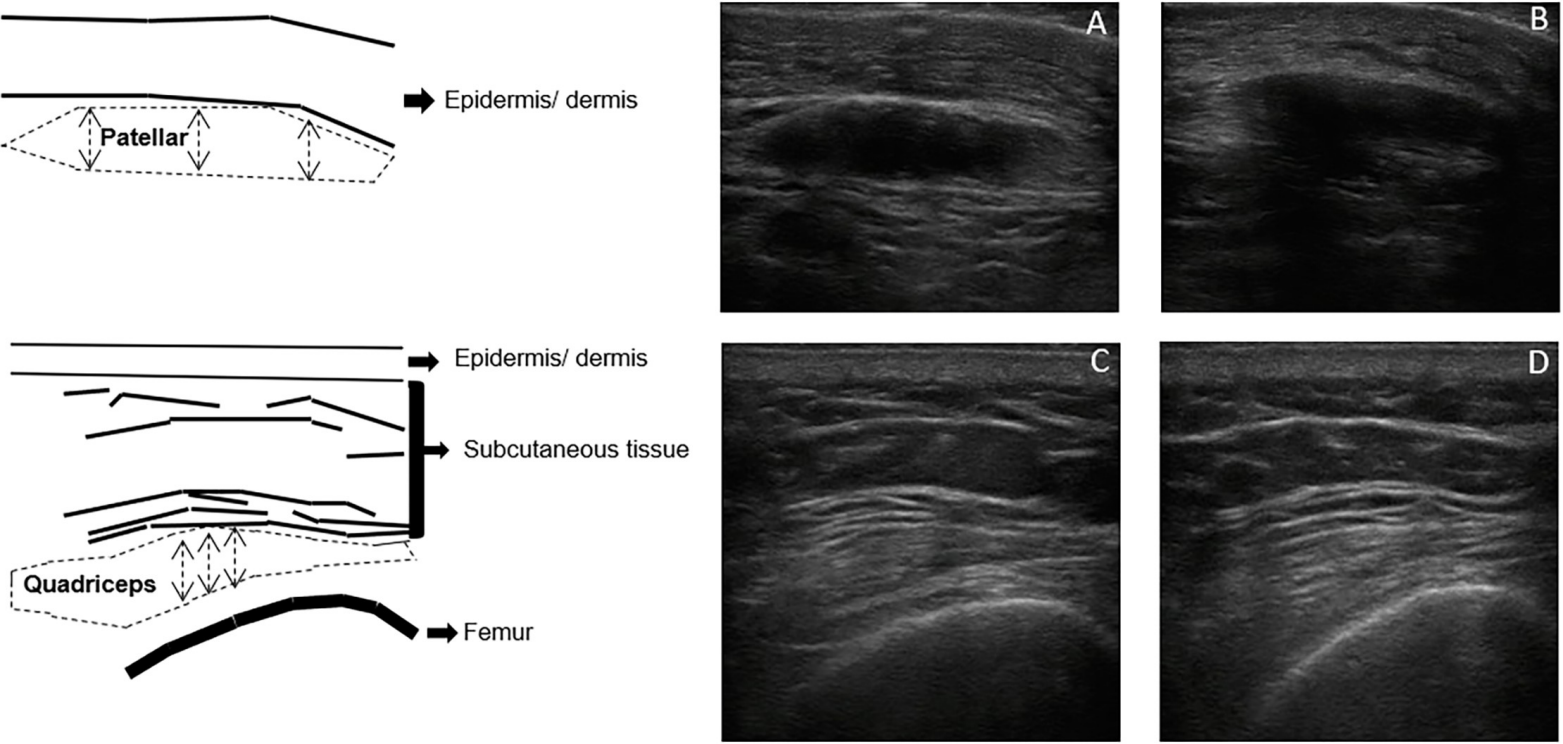
Sonographic appearance of patellar and quadriceps tendons. Example of ultrasonography scans of the patellar (upper part) and quadriceps (lower part) tendons. The left panel depicts the different structures schematically. The dotted line represents the region of interest for the cross-sectional area and echogenicity of each tendon. Tendon thickness is symbolized by the dotted arrows. Tendon scans were conducted with a 4.9 cm depth. For example, considering the qualitative analysis performed in this study, A and C were qualified as “good” images; B and D were qualified as “bad” images.

The experienced rater classified images as either: good–image with well-defined tendon borders–or bad–image with poorly-defined tendon borders. The opinions were employed in the inter-rater agreement analysis described below. The image quality analysis considered all landmarks; however, the average of the three measures obtained from the patellar tendon was calculated for the next analysis. The same image quality analysis was carried out at 2 different times, with a 7-day interval to verify the actual characterization of the image (Fig 1).

We calculated the thickness in three different locations along each image for both patellar and quadriceps tendons (Fig 1). The mean value was subsequently used for analyses. In turn, the CSA was measured by the trace technique consisting of the delimitation of the entire visible area of the tendon, excluding the peritendinous sheath (visible as a distinct, highly echogenic region both superficial and deep in the tendon) [19]. Echogenicity measurements were performed by the trace technique adopting the same area previously described for the calculation of CSA as the region of interest. Echogenicity was represented by a histogram on a gray scale with values ranging from 0 to 255 (0: black/no wave reflection; 255: white/total wave reflection).

For each outcome (thickness, CSA and echogenicity), first and second records were compared for each rater for patellar and quadriceps tendons, defining 6 experimental conditions for testing intra-rater reliability. For each variable, the analysis was carried out by the experienced and novice raters comparing single and double measures of the two tendons, defining a further 6 analyses to verify inter-rater reliability. The 6 experimental conditions of each analysis (intra and inter-rater) were divided by tendons (patellar and quadriceps) containing six Bland-Altman plots organized by pairs of repeated records in the columns (1^st^ versus 2^nd^ records for intra-rater or experienced versus novice measures for inter-rater) and by variables in the rows (thickness, CSA, and echogenicity). These measurements were performed twice consecutively by the experienced rater and the mean values considered for statistical analyses [34]. The intra-rater reliability was assessed between two subsequent repeated records taken by the raters (1^st^ and 2^nd^ records). For inter-rater analysis, the first and the average between the first and second repeated records defined the single and double measures, respectively.

### Statistical analysis

The Shapiro-Wilk normality test identified non-Gaussian and Gaussian distribution for the data recorded from patellar and quadriceps tendons respectively. For this reason, the repeated measures recorded from the patellar tendon are represented as median followed by the lower and upper limits of the 95% Confidence Interval (non-parametric analysis) whereas mean and Standard Deviation (SD) are used for the data recorded from the quadriceps tendon (parametric analysis). The Wilcoxon matched-pairs signed test was used for all comparisons. Statistical significance was accepted at p<0.05. Analysis was conducted using the first record (here called single) and the mean of the two records (double).

As mentioned, we considered two possibilities for the qualification of the image (good or bad), which were determined by the experienced examiner. The analysis of the agreement between experienced and novice records (inter-rater agreement) was performed using the Fleiss Kappa coefficient test applied for each region (proximal, middle, and distal regions) of the patellar tendon, and proximal region of the quadriceps tendon. We also calculated the gross agreement percentage (i.e., the percentage of observations in which the qualification of the records was the same). In accordance with Landis & Kock (1977), we considered values of kappa<0 as no agreement, 0>kappa≥0.20 (poor agreement), 0.20>kappa≥0.40 (fair agreement), 0.40>kappa≥0.60 (moderate agreement), 0.60>kappa>0.80 (substantial agreement), and 0.80≥kappa>1.00 (perfect agreement). A negative Kappa means that there is less agreement than would be expected by chance given the marginal distributions of ratings [35].

The intra and inter-rater reliabilities were processed for pairs of repeated measures; two images recorded by the same rater (1^st^ and 2^nd^ records) or two images recorded independently by different raters (experienced and novice). Initially, intraclass correlation coefficient (ICC) with a two-way random effects model either with single measure (ICC_2,1_) or with average measures (ICC_2,2_), calculated by taking an average of tha two raters’ measurements. Subsequently, the Bland-Altman (B&A) method was applied. The quality of the correlation detected by ICC was classified by the scale suggested by Lee et al (2012): 0 (absence), 0–0.19 (poor), 0.20–0.39 (weak), 0.40–0.59 (moderate), 0.60–0.79 (substantial), and ≥ 0.80 (almost complete) [36]. All statistical analyses were performed using SPSS for Mac (version 23, IBM, Chicago, Illinois, USA).

## Results

Twenty critically ill patients (90% male) admitted to the ICU (diagnosis/condition: neurosurgical, medical, and trauma) constituted the convenience sample of this study. On the day of evaluation, subjects had a median age of 48 (35–55) years, body mass index of 25 (23–28) kg/m^2^, median length of ICU stay of 11 (6–18) days, and duration of mechanical ventilation of 8 (4–18) days. The results of measurements of the patellar and quadriceps tendons did not present any significant differences between the experienced and novice raters or between single and double measurements for any of the reported variables (Table 1). On average, the experienced rater spent less time than the novice rater to acquire the ultrasound images (2.5 min vs 7.0 min, respectively).

**Table 1:** Measurement behaviour recorded in each of the 24 experimental conditions.

Experimental Conditions				Repeated Measures	
Raters	Tendons	Variables	Measures	Mean or Median	±SD or [min,max]
Experienced	Patellar	Thickness (cm)	single	0.36	[0.34,0.39]
			double	0.36	[0.33,0.39]
		CSA (cm^2^)	single	1.24	[1.10,1.37]
			double	1.24	[1.12,1.38]
		Echogenicity (au)	single	55.39	[49.29,60.37]
			double	55.28	[49.43,61.51]
	Quadriceps	Thickness (cm)	single	0.90	± 0.16
			double	0.90	± 0.16
		CSA (cm^2^)	single	2.68	± 0.54
			double	2.66	± 0.50
		Echogenicity (au)	single	52.68	± 12.95
			double	53.32	± 13.66
Novice	Patellar	Thickness (cm)	single	0.37	[0.33,0.41]
			double	0.37	[0.32,0.42]
		CSA (cm^2^)	single	1.34	[1.14,1.46]
			double	1.30	[1.12,1.48]
		Echogenicity (au)	single	59.35	[47.32,61.84]
			double	58.64	[44.42,62.20]
	Quadriceps	Thickness (cm)	single	0.90	± 0.18
			double	0.90	± 0.16
		CSA (cm^2^)	single	2.59	± 0.54
			double	2.59	± 0.54
		Echogenicity (au)	single	59.28	± 14.08
			double	58.86	± 14.09

Data recorded on the patellar tendon were obtained from twenty subjects (n = 20) whereas on the quadriceps tendon the sample was composed of sixteen (n = 16) due to the bad quality of the images. Data are presented as mean±SD (quadriceps tendon data) and median lower and upper limits of the 95% Confidence Interval (CI) of the median [min,max] (patellar tendon data). P > 0.05 for comparisons between experienced versus novice and single versus double for all reported variables. Abbreviations: CSA–Cross-sectional area; SD–Standard Deviation; min–minimum; max–maximum.

### Inter-rater agreement

The inter-rater analysis (Table 2) demonstrated that the majority of endpoints showed at least substantial agreement. The weakest agreement (k = 0.571) and the lowest gross agreement percentage (85%) were found for the second record for the quadriceps tendon considering statistically significant results. It should be noted that Kappa statistics were not computed in the middle of the patellar tendon length. No statistics were possible because all images in this tendon region were classified as “good”. There was no statistically significant agreement in the first record of quadriceps tendon and second record of the patellar distal region.

**Table 2:** Agreement between the quality of records acquired by two independent raters.

Ultrasound Images			Experienced *vs* Novice		
Image	Tendons	Regions	Kappa	95% CI	% Agreement
1^st^ record	Patellar	Proximal	1.000	[1.000,1.000]	100.00%
		Middle	Kappa could not be computed*		
		Distal	0.643	[0.006,1.000]	95.00%
	Quadriceps	Proximal	0.306	[-1.139,0.750]	75.00%
	2^nd^ record	Patellar	Proximal	1.000	[1.000,1.000]	100.00%
Middle			Kappa could not be computed*		
Distal			-0.053	[-0.125,0.020]	90.00%
Quadriceps		Proximal	0.571	[1.142,1.000]	85.00%

The experienced rater expressed its opinion (good or bad image) on the first (1^st^) and second (2^nd^) records. The asterisk (*) indicates the condition when the kappa could not be computed as all records were classified as good. Abbreviation: *vs*–*versus*.

### Intra- and inter-rater reliability

**Fig 2:**
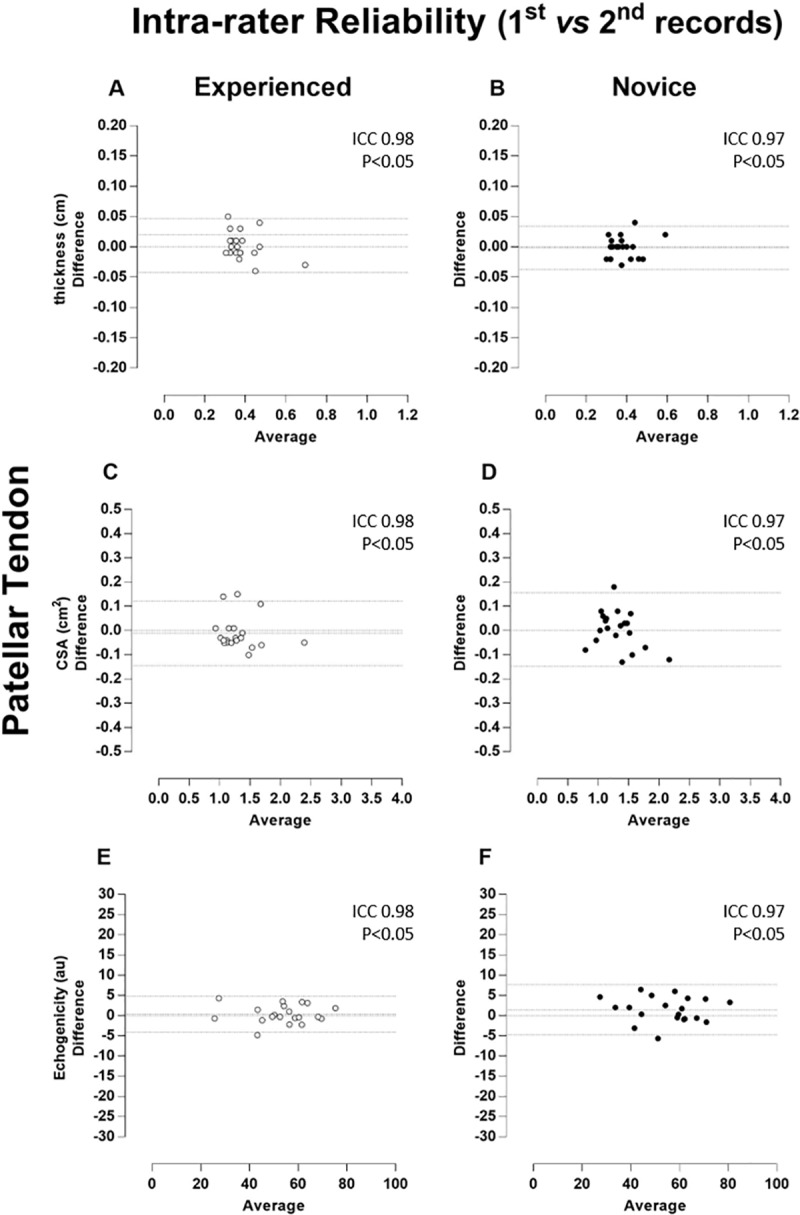
Patellar tendon–Intra-rater Reliability (1^st^ vs 2^nd^ records). Bland-Altman plots of the intra-rater comparison between first (1^st^) and second (2^nd^) repeated records of the thickness (A and B), cross-sectional area (CSA, C and D), and echogenicity (E and F) taken on the patellar tendon. The images taken by the experienced rater are in the left column (A, C, and E) and plotted by white circles, whereas those taken by the novice rater are in the right column (B, D, and F) and plotted by black circles. Besides the zero lines, the bias line and random error lines showing the 95% limits of agreement are represented by dashed lines.

**Fig 3:**
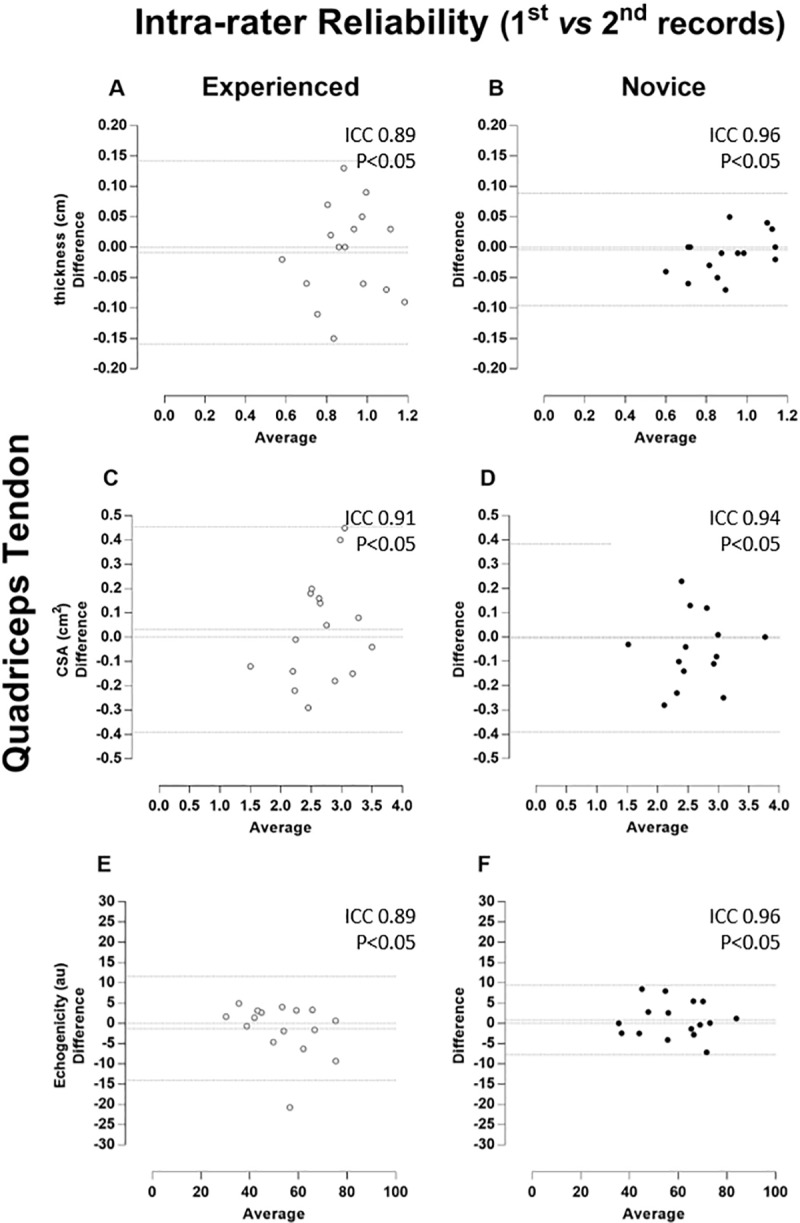
Quadriceps tendon–Intra-rater Reliability (1^st^ vs 2^nd^ records). Bland-Altman plots of the intra-rater comparison between first (1^st^) and second (2^nd^) repeated records of the thickness (A and B), cross-sectional area (CSA, C and D), and echogenicity (E and F) taken on the quadriceps tendon. The images taken by the experienced rater are in the left column (A, C, and E) and plotted by white circles, whereas those taken by the novice rater are in the right column (B, D, and F) and plotted by black circles. Besides the zero lines, the bias line and random error lines showing the 95% limits of agreement are represented by dashed lines.

**Fig 4:**
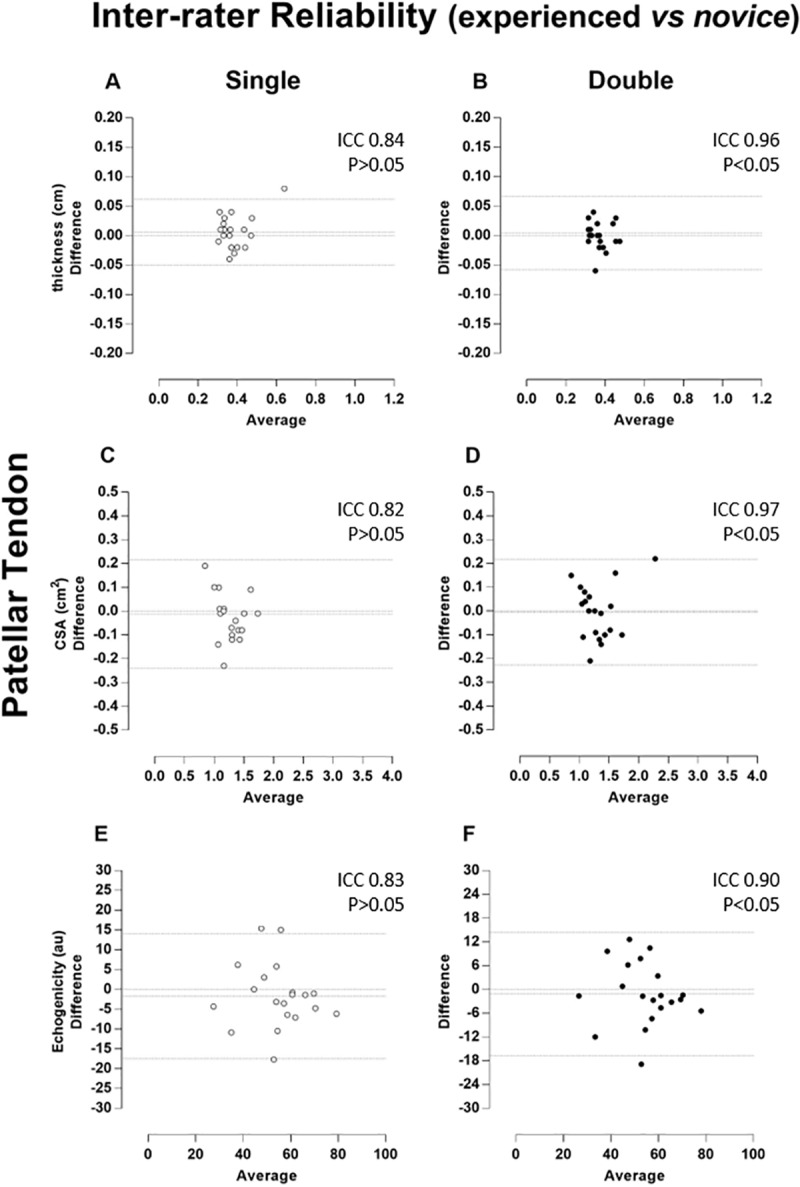
Patellar tendon–Inter-rater Reliability (experienced vs novice). Bland-Altman plots of the inter-rater comparison between repeated measures taken by experienced and novice raters, of the thickness (A and B), cross-sectional area (CSA, C and D), and echogenicity (E and F) on the patellar tendon. The single and double measures are respectively in the left (A, C, and E) and (B, D and F) right columns and plotted by white and black circles. Besides the zero lines, the bias line and random error lines showing the 95% limits of agreement are represented by dashed lines.

**Fig 5:**
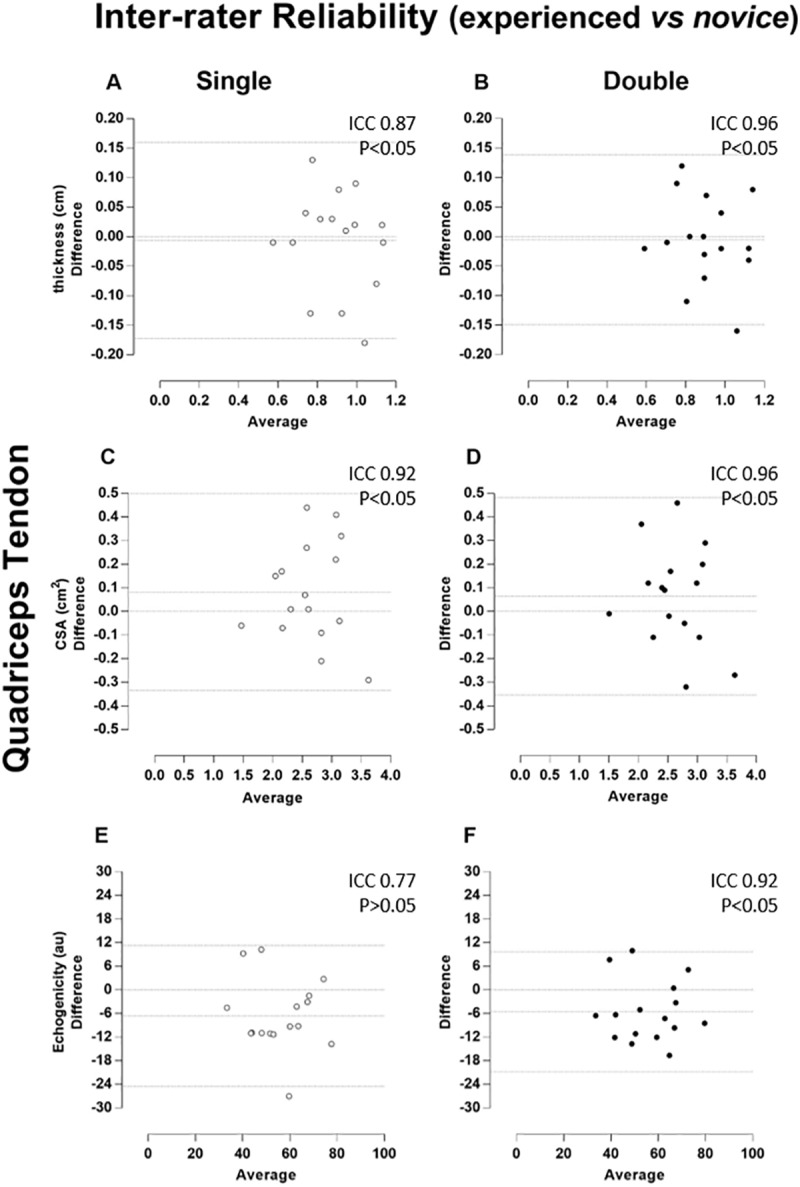
Quadriceps tendon–Inter-rater Reliability (experienced vs novice). Bland-Altman plots of the inter-rater comparison between repeated images taken by experienced and novice raters, of the thickness (A and B), cross-sectional area (CSA, C and D), and echogenicity (E and F) on the quadriceps tendon. The single and double measures are respectively in the left (A, C, and E) and (B, D, and F) right columns and plotted by white and black circles. Besides the zero line, the bias line and random error lines forming the 95% limits of agreement are presented by dashed lines.

The magnitude of the reliability (ICC) of tendon thickness, CSA and echogenicity was classified as “almost complete” no matter the rater. When considering inter-rater reliability, ICCs were classified as “almost complete” when two images were considered for analysis. However, patellar reliability revealed no statistically significant correlations using a single US image (p > 0.05). All measures of correlation are demonstrated together with Bland-Altman plots (Figs 2–5).

## Discussion

The present study confirmed the initial hypothesis demonstrating that the use of a standardized technique by experienced or novice raters resulted in reproducible measurements of tendon thickness, CSA, and echogenicity. Tendon imaging acquisition could be assessed by care providers with a minimal level of expertise in US devices. Thus, it may be possible to screen the effects of rehabilitative treatments on tendons.

The evaluation of US image quality through the kappa coefficient allowed us to establish the dimension of agreement beyond that expected by chance [37]. Agreement by chance can be demonstrated as gross agreement (% agreement in Table 2). For the distal patellar, there was a coincidence in the classification of 90% of the images acquired by different raters. However, this fact did not suggest statistical significance for the purpose of agreement analysis (is not a true agreement). It was observed that the statistically significant agreement was classified as moderate, reaching values of perfect agreement (κ = 1.0).

The inter-rater agreement on image quality showed that the middle region of the patellar tendon was the easiest region to obtain good quality images. However, the tendon distal region did not show statistical significance. The tendon borders in the distal region were more difficult to visualize, probably because of the tibial tuberosity. Coupling the US transducer to a prominent and rigid region at the extremities of the bones would have impaired complete image capture of the entire structure, possibly causing the borders to be unclear. Interestingly, the lowest agreement data were found for the quadriceps tendon. Tendon borders are less evident in the US images compared to patellar images. This may cause inconsistency in the classification of the image as being of good quality. Recently, the delimitation of the quadriceps tendon image was confirmed in the ultrasound image [30]. Despite being a crucial tendon for the knee extensor mechanism, this type of evaluation is still incipient, and improvement and familiarization with this method could result in greater consistency of image acquisition.

The ICC values of the patellar and quadriceps tendons for thickness, CSA and echogenicity reached a degree of correlation classified as "almost complete", even considering the novice rater. These data are in agreement with previous studies that analyzed the thickness and CSA by US in individuals with orthopedic lesions in the outpatient setting [19–22,26]. However, no studies have assessed tendon reliability in highly complex treatment ICU settings. Ekizos et al. [27] stated that US is not a reliable instrument for measuring the CSA of the patellar tendon of healthy young individuals. This discrepancy with our results can be justified by the different methods used for image acquisition. That study used the origin and insertion of the tendon (stable and easily visible points on the US), as well as the median (midway between the origin and insertion) and compared patellar tendon regions separately. In our study, the CSA was the average of three well-defined regions (25%, 50%, and 75% of tendon length) as previously proposed [6,31,38].

The Bland-Altman analysis showed that both repeated measures by the raters (intra-rater) and the repeated single and double measures between the raters (inter-rater) presented a predominant pattern of random variability. As the measurements were highly associated and limits of agreement (LOA) were small, the error was insignificant for most variables in both the patellar tendon and quadriceps tendon. This small error in repeated measures was described as a random type error associated with the A2 model of the B & A plot proposed by David and Giavarina [39]. In addition, there is no specific reason that can lead to this type of error.

Interestingly, intra-rater reliability seems to have a constant error in 3 groups of patients (5, 3, and 2 patients) and no error in 5 patients. The cases aligned in Fig 2B was named as the patient group. However, as the measures showed very high correlation (ICC 0.97), it is possible to suggest that there is no specific reason to induce this type of error, i.e., these results are associated with a random error. The quadriceps tendon was also an example of random error with almost complete ICC (Fig 3 - graphs A and B). It seems that the novice examiner chose the visual references for thickness and did not change this parameter due to the smaller LOA amplitude, in contrast to the experienced rater.

Unexpectedly, slightly lower ICC values were found for the experienced rater compared to the novice considering quadriceps tendon. The average time of examination by the novice rater was practically 3 times longer that of the experienced rater (7min vs. 2.5min), and it is possible to suggest that the longer time spent on image acquisition the better the image quality, implying more reliable quantification of the structure. In contrast, it was not surprising not found statistically significance in some single inter-rater condition. This could be due to the bias of inherent variability in different raters, which has been described previously [38,39].

It is important to note that there was no statistically significant when only one measure was used for some measurements (Figs 4 and 5). However, thickness, CSA, and echogenicity raw values did not show any significant differences. The greatest difference between absolute inter-examiner values was approximately 10% for the echogenicity measure (Table 1). Probably, this small difference did not reflect any significant clinical repercussion. In fact, echogenicity represents the tendon by a gray scale where black color is no wave reflection and white color refers to total wave reflection. Anisotropy is an artefact and occurs when organized fibrils may reflect a majority of the insonating sound beam in a direction away from the transducer. This will cause the tendon to change from brightly hyperechoic to darkly hypoechoic [12]. To prevent this unwanted effect, the ultrasound probe should be positioned perpendicular to the structure being imaged [13]. Since the images were blindly acquired by two raters, it is likely that the angulation of the transducer used in the evaluation was not exactly the same. Thus, there may be some discrepancy in the results of echogenicity. However, it is emphasized that a high correlation was found for echogenicity probably because it was used a well-defined and standardized acquisition protocol. This find is in agreement with another study performed on muscle [10].

To our knowledge, this is the first study on US reliability of the quadriceps tendon in critically ill patients. We found studies that confirmed the validity of the instrument for the diagnosis of traumatic injury [40,41], and established the dimensions of the tendon in immature patients [34], as well as a review study on the sonographic image of the quadriceps tendon [12]. The instrument’s ability to reproduce data should attest the use of this tool in both scientific and clinical research. Therefore, this study is the initial step to disseminating the use of US for serial evaluations of the quadriceps tendon in an intensive care setting.

### Study limitations

Further studies should develop strategies or devices to better understand the uniqueness of the US assessment. For example, reliability of the marking procedure itself (e.g., joint position) and determining image settings (e.g., depth/gain, transducer type) are still potential unintimated sources of error at the bedside. Besides, it should be noted that these results are applicable in the context of detecting longitudinal changes (where the same landmark is used over time). Further studies are required to determine the reproducibility of single time cross-sectional analysis, which involves the placement of a new landmark.

## Conclusions

Ultrasound imaging acquisition measures taken by independent raters for tendon thickness, CSA, and echogenicity monitoring of critically ill patients are acceptable and not influenced by rater experience. It may be important to take time for image acquisition to increase reproducibility.
